# Impact of improvement of sleep disturbance on symptoms and quality of life in patients with functional dyspepsia

**DOI:** 10.1186/s12876-021-01659-y

**Published:** 2021-02-18

**Authors:** Fumihiko Nakamura, Shiko Kuribayashi, Fumio Tanaka, Noriyuki Kawami, Yasuhiro Fujiwara, Katsuhiko Iwakiri, Motoyasu Kusano, Toshio Uraoka

**Affiliations:** 1 Department of Gastroenterology and Hepatology, Gunma University Graduate School of Medicine, 3-39-15 Showa-machi, Maebashi City, Gunma 371-8511 Japan; 2 Digestive Disease Center, Kohseichuo General Hospital, 1-11-7 Mita, Meguro-ku, Tokyo, 153-8581 Japan; 3 Department of Gastroenterology, Osaka City University Graduate School of Medicine, 1-5-7 Asahi-cho, Abeno-ku, Osaka, 545-8586 Japan; 4 Department of Gastroenterology, Nippon Medical School Graduate School of Medicine, 1-1-5 Sendagi, Bunkyo-ku, Tokyo, 113-8602 Japan

**Keywords:** Functional dyspepsia, Sleep disturbance, Sleep aids, Gastrointestinal symptoms, Quality of life

## Abstract

**Background/aims:**

Functional dyspepsia (FD) is often comorbid with sleep disturbance. However, it is not fully understood how sleep disturbance affects the pathophysiology of FD. We aimed to investigate the relationship between FD and sleep disturbance.

**Methods:**

We prospectively enrolled 20 FD patients with sleep disturbance between December 2018 and July 2019. Patients took sleep aids for 4 weeks and filled out questionnaires before and after taking sleep aids. Pittsburgh Sleep Quality Index (PSQI), Epworth Sleepiness Scale (ESS), and Athens Insomnia Scale (AIS) were used to evaluate the severity of their sleep disturbance. Modified Frequency Scale for the Symptoms of Gastroesophageal Reflux Disease (mFSSG), Gastrointestinal Symptom Rating Scale (GSRS), and the Japanese version of Patient Assessment of Constipation Quality of Life (JPAC-QOL) were used to evaluate the severity of GI symptoms. Short-Form 36-Item Health Survey (SF-36) was used to evaluate QOL. Pre- and post-sleep medication values of questionnaires were compared.

**Results:**

Among 20 enrolled patients, 16 completed the study protocol. Zolpidem, eszopiclone, and suvorexant were administered to six, nine, and one patient, respectively. Each median total score of questionnaires (pre-/post-sleep medication, respectively) was as follows: PSQI, 10.0/8.5; ESS, 12.5/5.0; AIS, 10.0/4.0; mFSSG, 21.0/16.0; GSRS, 44.0/31.0 (Pain in GSRS, 11.0/5.0); JPAC-QOL, 26.0/15.5; SF-36, 63.9/71.9. All of these results showed statistically significant differences between pre- and post-sleep medication (*p* < 0.05).

**Conclusions:**

Improvement of sleep disturbance by administration of sleep aids resulted in improvement of GI symptoms and QOL in patients with FD. This effect may be related to pain modification.

## Introduction

It is well known that many patients with functional gastrointestinal (GI) disorders have sleep disturbance [1–3]. Functional dyspepsia (FD) is a functional GI disease that is also frequently comorbid with sleep disturbance [4, 5]. However, most studies have just pointed out an association between GI symptoms and sleep disturbance, and only a few studies have focused on how GI symptoms change by improvement of sleep disturbance. As with gastroesophageal reflux disease (GERD) and irritable bowel syndrome (IBS), melatonin potentially improves symptoms in adult patients with FD [6]. However, the effect of melatonin on FD could not be confirmed in pediatric patients [7]. It is unclear how melatonin can improve symptoms of FD in adult patients and why such an effect could not be seen in pediatric patients. In addition, other kinds of sleep aids have not been evaluated in terms of relationship between FD symptoms and sleep disturbance. Thus, how sleep disturbance is related to the pathophysiology of FD remains unclear.

In terms of pathogenesis of GI symptoms caused by sleep disturbance in patients with GI disorders, Schey et al. showed that sleep disturbance can induce esophageal hypersensitivity in patients with GERD [8], and that PPIs can improve esophageal hypersensitivity induced by acid reflux events. In fact, ramelteon, a melatonin receptor agonist, has been reported to improve GERD-related as well as sleep-related symptoms [9]. Therefore, sleep disturbance could cause visceral hypersensitivity in patients with FD.

The aims of this study were to investigate the effect of sleep medication on GI symptoms in patients with FD and to clarify the relationship between FD and sleep disturbance.

## Materials and methods

### Subjects

A total of 20 consecutive patients with both FD and sleep disturbance were enrolled prospectively. All patients met the criteria of Rome IV for Functional Dyspepsia [10]. All patients had sleep disturbance with a total score of more than 5.5 in the Pittsburgh Sleep Quality Index (PSQI) [11]. Patients who had a history of GI surgery, those who had taken sleep aids, those who had changed therapy for FD except sleep aids during the study period, and those who were younger than 20 years old were excluded from the present study.

All patients gave written informed consent for this study, which was performed in accordance with the Helsinki Declaration. The study protocol was approved by the Institutional Review Board at Gunma University Hospital, Kohseichuo General Hospital and Osaka City University Graduate School of Medicine.

### Questionnaires

All patients were asked to fill out questionnaires to assess sleep quality, GI symptoms, mental illness, and quality of life (QOL).

#### Pittsburgh Sleep Quality Index

Sleep disturbance was evaluated by the Pittsburgh Sleep Quality Index (PSQI) [12], which consists of 17 individual items grouped into the following seven components: subjective sleep quality, sleep latency, sleep duration, habitual sleep efficacy, sleep difficulty, hypnotic use, and daytime dysfunction. Each category receives a score of 0–3 (max total score 21). A higher total score indicates poorer sleep quality. The Japanese version of the PSQI has been validated and was used in the present study [11]. A total PSQI score of more than 5.5 was considered to indicate the presence of sleep disturbance [11].

#### Epworth Sleepiness Scale

The Epworth Sleepiness Scale (ESS) is a self-report instrument for measuring a patient’s daytime sleepiness [13]. It consists of eight questions, with a 4-point scale (scored 0–3) being used for each question. The total score is the sum of points from each question (i.e., 0–24) with a higher score indicating stronger subjective daytime sleepiness. The Japanese version of the ESS (JESS) has been validated and was used in the present study [14].

#### Athens Insomnia Scale

The Athens Insomnia Scale (AIS) was created to assess the severity of insomnia on the basis of the ICD-10 diagnostic criteria for insomnia [15]. The AIS consists of eight items rated on a 4-point scale (0 = on problem at all, 1 = slightly problematic, 2 = markedly problematic, 3 = extremely problematic). The cutoff score used to identify pathological insomnia was previously determined as a score of 6 points [16]. The Japanese version of the AIS has been validated and was used in the present study [17].

#### Modified Frequency Scale for the Symptoms of GERD

The Frequency Scale for the Symptoms of GERD (FSSG) is a questionnaire used to diagnose GERD and assess the response to treatment [18]. The modified FSSG (mFSSG) can assess both reflux-related and dyspepsia-related symptoms [19]. The mFSSG consists of 14 items, divided into seven items relating to reflux symptoms and seven relating to dyspepsia symptoms. A higher total score indicates more severe GERD or dyspepsia-related symptoms. We utilized the mFSSG in the present study.

#### Gastrointestinal Symptom Rating Scale

The Gastrointestinal Symptom Rating Scale (GSRS) is used to evaluate general GI symptoms and consists of 15 items rated on a 7-point Likert scale [20]. The 15 items cover five subscales (reflux, abdominal pain, indigestion, diarrhea, and constipation).

#### Constipation Scoring System

The Constipation Scoring System (CSS) is used to evaluate the prevalence and severity of constipation [21]. The scoring system contains eight variables: frequency of bowel movement, difficult or painful evacuation, completeness of evacuation, abdominal pain, time per attempt, use and type of assistance (including laxatives, digitations, or enemas), number of unsuccessful attempts at evacuation in a 24-h period, and duration of constipation. The CSS consists of seven items scored on a 5-point Likert scale from 0 (none of the time) to 4 (all of the time), and one item (use and type of assistance) rated on a 0–2 scale. The total score can range from 0 (normal) to 30 (severe constipation), and a cutoff score of 15 suggests constipation.

#### Patient Assessment of Constipation Quality of Life

Patient Assessment of Constipation Quality of Life (PAC-QOL) is used to measure the QOL in patients with constipation [22]. The PAC-QOL consists of 28 items grouped into four subscales (physical discomfort, psychosocial discomfort, worries and concerns, and satisfaction). The first three subscales provide a patient dissatisfaction index, with an overall score from 0 to 96 (lower scores correspond to better QOL). The satisfaction subscale includes four items with a global score from 0 to 16. Each patient’s self-reported definitive outcome is defined as poor (0–4), fairly good (5–8), good (9–12), or excellent (13–16). The Japanese version of the PAC-QOL (JPAC-QOL) has been validated and was used in the present study [23].

#### Hospital Anxiety and Depression Scale

The Hospital Anxiety and Depression Scale (HADS) is used to evaluate anxiety and depression [24]. HADS is divided into an anxiety subscale (HADS-A) and a depression subscale (HADS-D). Each subscale contains seven items that are scored from 0 to 3, which give a total subscale score from 0 to 21. A higher HADS score indicates that the person is more depressive or more anxious. The Japanese version of the HADS has been validated and was used in the present study [25].

#### Medical Outcome Trust 36-Item Short-Form Health Survey

The Medical Outcome Trust 36-Item Short-Form Health Survey (SF-36) is used to evaluate health-related QOL in general. Items contribute toward assessment of eight components: physical functioning (PF), role physical (RP), bodily pain (BP), general health (GH), vitality (VT), social functioning (SF), role emotional (RE), and mental health (MH). The Japanese version of the SF-36 has been validated and was used in the present study [26].

### Study protocol

Patients were asked to fill out their questionnaires before and 4 weeks after taking sleep aids. Zolpidem, suvorexant, or eszopiclone were administered as sleep aids. Although acid-secretion inhibitors or prokinetics had already been administered in almost half of the patients, during the study period treatments for FD were not changed.

### Sleep aids

There are several types of insomnia such as sleep onset insomnia, sleep maintenance insomnia and insomnia with early morning awakening. Zolpidem is used for sleep onset insomnia and eszopiclone and suvorexant can be used for both sleep onset insomnia and sleep maintenance insomnia [27]. The potential for abuse and adverse events may be lower with suvorexant than with benzodiazepine receptor agonists [27–29]. Therefore, in this study the attending physician evaluated and selected the best sleep aid for the patients based on types of insomnia, patient age and comorbidities. The doses of these sleep aids were determined according to the Japanese package inserts of the drugs. Among the 16 patients, zolpidem, eszopiclone, and suvorexant were administered for 4 weeks in six, nine, and one patient, respectively.

### Primary and secondary outcomes

The primary outcomes of the study were whether or not the average change in dyspepsia scores in mFSSG had improved by 40% by administration of sleep aids, and whether the scores in mFSSG were significantly decreased by administration of sleep aids. The secondary outcomes were changes in other gastrointestinal symptoms, quality of life and depression mode. In addition, presence and improvement of sleep disturbance were confirmed by three kinds of validated questionnaires. It is known that many patients with FD have also other functional diseases, such as IBS or chronic constipation. Therefore, changes in other GI symptoms, such as constipation or diarrhea, were also evaluated.

### Sample size

Jha et al. reported that the score of reflux symptoms in GSRS in patients with GERD were improved by 40% with administration of ramelteon (8 mg per day) for 4 weeks; therefore, we have assumed that dyspepsia symptoms in mFSSG in patients with FD will also be improved by 40% with sleep aids [9]. With a standard deviation of 0.5, a power of 80%, and level of significance of 5% using the paired t-test, it was calculated that a sample size of 15 is needed. A total of 25% of patients could be excluded from the study, meaning that 20 patients were required for the purposes of the present study.

### Statistical analysis

Data in the text are presented as medians and interquartile range (IQR) unless stated otherwise. The Wilcoxon signed-rank test was used for comparing scores in questionnaires before and 4 weeks after taking sleep aids as appropriate. A *p* value of less than 0.05 was considered statistically significant. Statistical analyses were performed by SPSS Statistics ver. 25 (IBM, Armonk, NY, USA).

## Results

### Patients’ characteristics

Of the 20 patients in total, four were excluded from the analysis. One patient had excessive sleepiness after talking a sleep aid and thus could not tolerate taking it for 4 weeks. Three patients refused to take a sleep aid after agreeing to participate in the study. Thus, a total of 16 patients were evaluated in the present study. All analyzed subjects were cared for by an author (FN).

Of these 16 patients, nine were classified with post-prandial distress syndrome (PDS) and seven with epigastric pain syndrome (EPS). Nine patients had symptoms related to non-erosive GERD (NERD), IBS and chronic constipation. They were classified as having overlap syndrome. Four patients had constipation; however, none had diarrhea. Eight patients took acid-secretion inhibitors and 10 took prokinetics before and during the study; these medications were not changed during the study (Table 1).

**Table 1:** Baseline patient’s characteristics and breakdown of the concomitant drugs

	FD Number of patients. (%)
*Baseline patient’s characteristics*	
Total number of patients	16
Age, median [IQR], years old	57 [42, 73]
Male:female	6:10
Subtypes of FD	
PDS	9 (47.4)
EPS	7 (52.6)
Overlap symptoms	
With NERD	5 (31.3)
With IBS-C	3 (18.8)
With chronic constipation	1 (6.3)
*Concomitant drugs*	
Acid secretion inhibitor	
Esomeprazole magnesium hydrate	5 (31.3)
Vonoprazan fumarate	3 (18.8)
Prokinetic agents	
Itoplide hydrochloride	2 (12.5)
Acotiamide hydrochloride hydrate	1 (6.3)
Herbal medicine	
Rikkunshito	6 (37.5)
Laxative	
Magnesium oxide	1 (6.3)
Stool stabilizer	
Polycarbophil calcium	1 (6.3)

*FD* functional dyspepsia, *PDS* post-prandial distress syndrome, *EPS* epigastric pain syndrome, *NERD* non-erosive reflux disease, *IBS-C* irritable bowel syndrome with constipation*, IQR* interquartile range

### Effect of sleep aids on sleep disturbance

Sleep disturbance was significantly improved by 4-week administration of sleep aids. Median total scores changed from 10.0 to 8.5 points in PSQI, 12.5 to 5.0 points in ESS, and 10.0 to 4.0 points in AIS, respectively (*p* < 0.05) (Table 2). Although the PSQI contains an item regarding the frequency of taking sleep aids (more than three times per a week = 3 points), the scores after taking sleep aids, even including the item (3 points), were significantly lower than those before taking sleep aids (*p* < 0.05). Therefore, sleep condition became almost normal after sleep aids administration in PSQI.

**Table 2:** Results of sleep related questionnnaire

		FD (n = 16)			
	Score, Median [IQR]			Variability rates, (%), Median [IQR]	*p* value
	Before taking sleep aids		After taking sleep aids		
*Sleep related questionnaires*					
Pittsburgh Sleep Quality Index (PSQI)	10.0 [8.3, 13.0]		8.5 [7.0, 10.0]	− 18.4 [− 27.7, 0]	.015
Epworth Sleepiness Scale (ESS)	12.5 [8.3, 15.0]		5.0 [4.0, 7.0]	− 53.3 [− 66.7, − 42.0]	< .001
Athens Insomnia Scale (AIS)	10.0 [7.3, 13.0]		4.0 [3.0, 7.8]	− 48.1 [− 81.2, − 27.2]	.006

*FD* functional dyspepsia, *IQR* interquartile range

### Effect of sleep aids on GI symptoms

The average change in dyspepsia scores and the 95% confidence interval for difference of means in mFSSG by sleep aids administration was − 37.8% [− 63.4, − 12.2] (*p* < 0.01). Since data was not normally distributed, the median change was − 44.1% (Table 3).

**Table 3:** Results of each questionnaire on gastrointestinal symptoms

	FD (n = 16)			
	Score, Median [IQR]		Variability rates, (%), Median [IQR]	*p* value
	Before taking sleep aids	After taking sleep aids		
*Gastrointestinal symptoms*				
Modified Frequency Scale for the Symptoms of GERD (mFSSG)				
Total score	21.0 [17.0, 30.5]	16.0 [6.0, 19.0]	− 34.7 [− 66.8, + 4.2]	.004
Reflux score	8.0 [5.5, 14.5]	6.0 [3.3, 8.8]	− 38.8 [− 62.7, − 3.6]	.011
Dyspepsia score	12.0 [9.0, 17.8]	8.5 [2.0, 13.0]	− 44.1 [− 79.2, − 3.6]	.005
Gastrointestinal Symptom Rating Scale (GSRS)				
Total Score	44.0 [37.5, 54.8]	31.0 [22.0, 40.5]	− 31.4 [− 46.0, − 22.3]	.005
Reflux	6.5 [3.5, 9.8]	4.0 [2.0, 6.0]	− 36.7 [− 57.5, 0]	.012
Abdominal pain	11.0 [6.3, 14.8]	5.0 [3.0, 9.0]	− 45.0 [− 66.7, − 11.8]	.002
Indigestion	11.0 [8.3, 15.8]	8.0 [5.3, 10.8]	− 34.5 [− 41.6, − 16.0]	.004
Diarrhea	7.0 [3.0, 9.8]	6.5 [4.0, 8.8]	0 [− 29.5, 0]	.199
Constipation	7.5 [5.3, 11.0]	6.0 [4.0, 8.8]	− 26.2 [− 40.0, − 2.3]	.020
Constipation scoring system (CSS)	6.0 [4.3, 10.8]	6.0 [3.0, 9.5]	− 10.0 [− 39.4, + 6.2]	.166
Japanese Version of the Patient Assessment of Constipation Quality of Life (JPAC− QOL)				
Total score	26.0 [17.5, 56.8]	15.5 [9.0, 42.5]	− 30.4 [− 65.3, 0]	.005
Physical discomfort	6.0 [4.3, 8.0]	3.5 [2.3, 8.0]	− 41.7 [− 50.0, 0]	.009
Psychological discomfort	5.5 [4.0, 10.8]	6.0 [2.0, 10.8]	− 18.3 [− 50.0, 0]	.030
Worries and concerns	9.5 [6.0, 20.8]	5.0 [0.3, 18.3]	− 33.1 [− 98.1, − 1.5]	.009
Satisfaction	7.0 [2.0, 13.8]	5.0 [0, 7.0]	− 42.8 [− 92.9, − 1.9]	.012

*FD* functional dyspepsia, *GERD* gastroesophageal reflux disease, *IQR* interquartile range

**Fig. 1:**
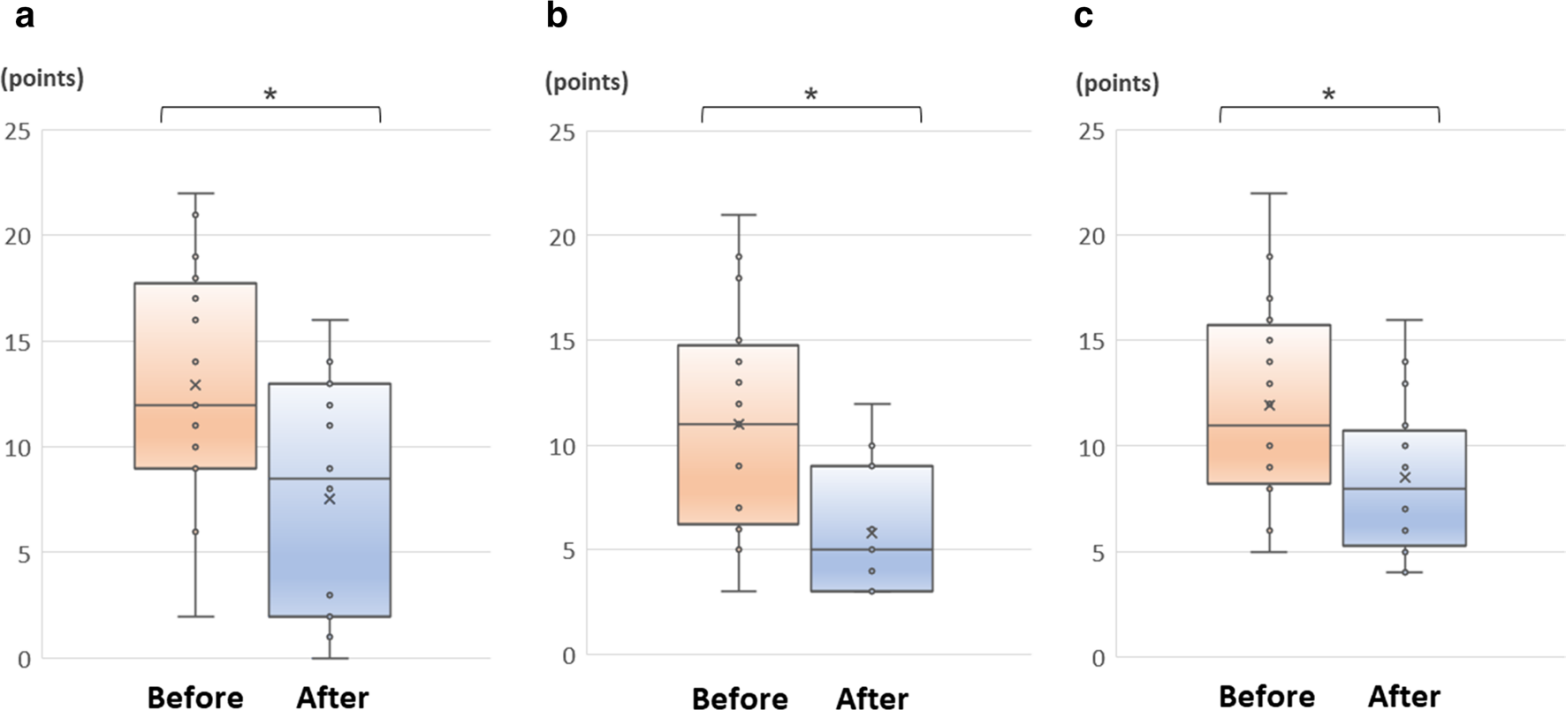
Variations of questionnaire score on FD symptoms. **a** mFSSG (Dyspepsia Score); **b** GSRS (Abdominal Pain); **c** GSRS (Indigestion). Orange and blue boxplot reflect the scores before and after taking sleep aids, respectively (n = 16). Statistical analysis was performed by using the Wilcoxon signed-rank test. *p < 0.05. *FD* functional dyspepsia, *mFSSG* modified frequency scale for the symptoms of gastroesophageal reflux disease, *GSRS* gastrointestinal symptom rating scale

Other GI symptoms were also significantly improved by a 4-week administration of sleep aids. The median total scores showed statistically significant improvement from 21.0 to 16.0 points in mFSSG and from 44.0 to 31.0 points in GSRS (*p* < 0.05). Reflux and dyspepsiascores in mFSSG, and reflux, abdominal pain, and indigestion symptoms in GSRS were significantly improved (*p* < 0.05) (Table 3) (Fig. 1). The total score of CSS was unchanged. The median total score for the JPAC-QOL after administration of sleep aids improved: all subscale items showed improved scores after 4 weeks of sleep aids (*p* < 0.05) (Table 3).

### Effect of sleep aids on psychological condition

Psychological status was significantly improved by a 4-week administration of sleep aids. The HADS total score showed a statistically significant improvement from 13.0 to 12.5 points after taking sleep aids (*p* < 0.05) (Table 4). Although depression scores did not change, anxiety scores were improved significantly (*p* < 0.05).

**Table 4:** Results of each questionnaire on psychological condition and QOL

	FD (n = 16)			
	Score, Median [IQR]		Variability rates, (%), Median [IQR]	*p* value
	Before taking sleep aids	After taking sleep aids		
*Psychological condition*				
Hospital Anxiety and Depression Scale (HADS)				
Total Score	13.0 [10.3, 19.8]	12.5 [6.3, 17.0]	− 15.9 [− 37.6, + 12.5]	.030
HADS-A	7.5 [6.0, 10.5]	6.0 [4.0, 9.8]	− 9.6 [− 42.2, 0]	.020
HADS-D	6.0 [4.0, 9.8]	5.5 [3.0, 8.8]	− 23.6 [− 33.3, + 12.5]	.140
*QOL*				
The Short-Form 36-Item Health Survey (SF-36)				
Total score	63.9 [42.3, 71.2]	71.9 [58.3, 77.3]	+ 5.0 [0, + 33.6]	.017
Physical functioning (PF)	80.0 [70.0, 90.0]	90.0 [75.0, 100.0]	+ 5.3 [0, + 12.5]	.011
Role physical (RP)	75.0 [56.3, 100.0]	75.0 [62.5, 93.8]	0 [− 11.2, + 50.0]	.556
Bodily Pain (BP)	41.0 [22.0, 52.0]	52.0 [41.0, 72.0]	+ 35.5 [+ 19.6, + 86.4]	.001
General health (GH)	45.0 [15.0, 57.0]	45.0 [35.0, 67.0]	+ 21.8 [0, + 66.7]	.007
Vitality (VT)	43.8 [31.3, 56.3]	50.0 [37.5, 62.5]	+ 11.0 [− 16.5, + 50.0]	.306
Social functioning (SF)	75.0 [37.5, 75.0]	87.5 [62.5, 100.0]	+ 16.7 [0, + 33.3]	.009
Role emotional (RE)	83.3 [58.3, 100.0]	91.7 [66.7, 100.0]	0 [− 16.6, + 42.9]	.207
Mental health (MH)	60.0 [50.0, 75.0]	65.0 [50.0, 80.0]	0 [0, + 33.3]	.282

*FD* functional dyspepsia, *HADS-A* hospital anxiety and depression scale- anxiety subscale, *HADS-D* hospital anxiety and depression scale- depression subscale, *QOL* quality of life, *IQR* interquartile range

### Effect of sleep aids on QOL

The baseline scores for BP, GH, and VT showed lower scores than the other subscale items. SF-36 showed statistically significant improvement of the median total score from 63.9 to 71.9 points after 4 weeks of taking sleep aids (*p* < 0.05) (Table 4). The median scores for PF, BP, and SF also showed statistically significant improvement. Although the GH scores were significantly different after 4-week administration of sleep aids, the median score was unchanged.

### *Helicobacter pylori* infection

*Helicobacter pylori* infection is an important pathogenesis of FD [30]. Since all patients underwent esophagogastroduodenoscopy (EGD) before the clinical diagnosis of FD, we checked the presence of gastric atrophy [31–33] in their stomach and their histories of *H. pylori* eradication. Out of 16 patients 12 patients did not have gastric atrophy, which indicates that these patients did not have *H. pylori* infection. Although the rest of the patients had gastric atrophy, three patients had received eradication therapy several years ago. The titer of immunoglobulin G for *H. pylori* in one patient with gastric atrophy without eradication therapy denied *H. pylori* infection. None of the patients had undergone eradication therapy within one year of the study enrollment; therefore, *H. pylori* infection would not affect the results of the study. Additionally, gastritis, gastric ulcer and other organic diseases were not detected in all patients.

## Discussion

This study revealed the effects and clinical impacts of improved sleep disturbance on GI symptoms in patients with FD. Improved sleep disturbance by administration of sleep aids resulted in improved GI symptoms, anxiety, and QOL. It is interesting that the use of sleep-inducing drugs was associated with reduced pain as well as improvement of dyspeptic symptoms in FD patients. Therefore, it is important to evaluate whether a patient with FD also has sleep disturbance. If this is the case, sleep aids could improve the GI symptoms in addition to sleep disturbance.

All sleep aids used in the present study could affect gastrointestinal motility, gastric secretion and mucosal secretion directly. In addition, these drugs could also affect patients’ nociception directly. γ-aminobutyric acid (GABA) receptor agonists could affect vagally mediated gastric acid secretion [34–39], mucosal secretion [40] and gastrointestinal motility [41, 42]. GABA type A (GABA_A_) receptor agonists and GABA type B (GABA_B_) receptor agonists could have opposite effects on gastric acid secretion. GABA_A_ receptor agonists could increase gastric acid secretion, while GABA_B_ receptor agonists could decrease it [39]. Therefore, zolpidem and eszopiclone, GABA_A_ receptor agonists, could increase vagally mediated gastric acid secretion, and increased gastric acid secretion may worsen gastrointestinal symptoms in patients with FD. However, these drugs have also antinociception effects. Although most reports showed a relationship between somatic pain and GABA_A_ receptors [43–45], the involvement in visceral pain has also been confirmed [46]. Thus, these drugs per se might improve symptoms in patients with FD. Regarding effects of suvorexant on gastrointestinal symptoms, orexin could regulate mucosal secretion [47], and could increase gastric acid secretion [48] and gastrointestinal motility [49–51]. An orexin receptor antagonist, suvorexant, may therefore inhibit gastric acid secretion, which could improve patients’ symptoms. However, orexin is also involved in stress-induced analgesia [52–54] and is involved in antinociceptive effects on visceral perception [55]. Therefore, suvorexant could worsen patients’ symptoms. In fact, gastrointestinal symptoms in the patient receiving suvorexant in the present study were worsened after suvorexant administration. However, clinical trials with suvorexant showed that side effects of suvorexant associated with gastrointestinal symptoms have been infrequent [56]. It is necessary to confirm the effects of suvorexant on gastrointestinal symptoms in patients with FD by further studies. When the patient receiving suvorexant was excluded from the study, the results, except those related to anxiety, were not changed. The total HADS score was also decreased, if the patient on suvorexant was excluded; however, the difference did not reach statistical significance.

In terms of the pathogenesis of symptoms in patients with FD, sleep disturbance itself could cause chronic pain [57] and visceral hypersensitivity [8]. Our data cannot address whether improvement of sleep disturbance could result in improvement of GI symptoms, whether sleep aids per se could cause the improvement, or whether both factors could contribute to the improvement.

It is known that the incidence of behavioral abnormalities and sleep disturbance is higher in patients with FD than those without FD [2]. Several reports have shown that cognitive or behavioral therapy was effective in patients with FD [58, 59] as well as sleep disturbance [60]. Although there is no study in which effects of cognitive behavioral therapy for insomnia on GI symptoms are evaluated, improvement of sleep disturbance by cognitive behavioral therapy may reduce GI symptoms in FD patients with sleep disturbance if improvement of sleep disturbance, not the direct effect of sleep aids, is important for improvement of GI symptoms. In addition, cognitive behavioral therapy for both insomnia and FD could be applied. Since cognitive sleep therapy does not have any adverse effect, cognitive behavioral therapy could be performed in FD patients with sleep disturbance before sleep aids administration.

Gender differences may exist in the pathophysiology of FD [61, 62]. Although little is known, gender differences in gastric emptying [63], visceral perception [64–66], ghrelin [67, 68] and altered functional connectivity of the amygdala [69] have been proposed to be involved in the pathophysiology of FD. When scores were compared between genders in the present study, total scores of PSQI, reflux symptoms in mFSSG and total scores in HADS were significantly more improved in females than in males. However, other scores were not different between the two groups. Nevertheless, the number of patients may not be sufficient to evaluate the gender differences. In addition, we did not check the status of menopause in female patients. Further studies are required to address this point.

Although there were significant improvements in patients’ symptoms over the study period, their GI symptoms did not completely disappear. Thus, it is necessary to continue treatment with standard therapeutic agents for FD if sleep aids are used for sleep disturbance.

Interestingly, the scores for not only FD symptoms but also reflux and constipation symptoms in the mFSSG, GSRS, and JPAC-QOL were also significantly improved after administration of sleep aids. Many patients with FD have other GI disorders, such as GERD, chronic constipation or IBS. Regarding reflux symptoms, it is known that sleep disturbance may induce esophageal hypersensitivity [9]. The improvement of reflux symptoms in patients with FD overlapped with GERD could be related to pain modulation by sleep aids. Regarding constipation, the CSS includes items involved bowel habits as well as symptoms. The number of questions related to bowel habits is much higher than the number of questions related to symptoms; therefore, the CSS can be used to evaluate bowel habits rather than symptoms. Total scores in the CSS were not changed, which indicated that bowel habits were not changed after administration of sleep aids. However, symptoms related to constipation were significantly improved. Only four patients in this study had constipation, which may be an inadequate number of patients for analysis. However, it is known that some patients with hidden constipation symptoms do not recognize that they have constipation [70]. If this kind of patients were present in the study, the effects of sleep aids on constipation might have been related to administration of sleep aids or to placebo effects. Further studies are required to confirm these results.

The present study has several limitations. First, this was a single-arm study and placebo agents were not used. This is important because symptoms attributable to a functional GI disorder such as FD could be affected by placebo sleep aids in terms of treatment. Therefore, randomized controlled trial should be planned in the future to confirm our results. Second, only zolpidem, eszopiclone, and suvorexant were used as sleep aids, and comparison with melatonin was not conducted. Third, the study included a small number of patients, although the sample was sufficient to validate the primary endpoint according to the calculation of sample size. We were unable to compare EPS with PDS and to analyze causal relationships between improvement of GI symptoms and other factors using a regression analysis because of the small number of cases. In addition, there were two kinds of patients: patients with FD who did not receive the standard therapy and those who had refractory symptoms against the standard therapy. Furthermore, all patients analyzed in the study received care by one physician only. The relationship between patient and physician could affect study outcomes. Further multicenter studies involving a larger number of cases and enrolling FD patients without the standard therapy are necessary. Fourth, all questionnaires were only completed before taking sleep aids and after a 4-week administration of these agents. The long-term outcomes after administration of sleep aids therefore remain unclear and should be investigated in future studies. Finally, only FD patients with sleep disturbance were enrolled in the study. It is not known whether sleep aids could have additional effects on FD patients without sleep disturbance.

## Conclusions

Improvement of sleep disturbance by sleep aids administration resulted in improvement of GI symptoms, anxiety, and QOL in patients with FD. These effects may be related to pain modification.

## Data Availability

The datasets analyzed during the current study are available from the corresponding author on reasonable request.

## Abbreviations

FD: Functional dyspepsia
PDS: Post-prandial distress syndrome
EPS: Epigastric pain syndrome
GERD: Gastroesophageal reflux disease
NERD: Non-erosive reflux disease
PPI: Proton-pump inhibitor
IBS: Irritable bowel syndrome
QOL: Quality of life
PSQI: Pittsburgh Sleep Quality Index
ESS: Epworth Sleepiness Scale
AIS: Athens Insomnia Scale
mFSSG: Modified Frequency Scale for the Symptoms of Gastroesophageal Reflux Disease
GSRS: Gastrointestinal Symptom Rating Scale
CSS: Constipation Scoring System
JPAC-QOL: Japanese version of the Patient Assessment of Constipation Quality of Life
HADS: Hospital Anxiety and Depression Scale
SF-36: Short-form 36-Item Health Survey
HRQOL: Health-Related Quality of Life
PF: Physical functioning
RP: Role physical
BP: Bodily pain
GH: General health
VT: Vitality
SF: Social functioning
RE: Role emotional
MH: Mental health
IQR: Interquartile range
